# Large nitrous oxide emissions from arable soils after crop harvests prior to sowing

**DOI:** 10.1007/s10705-024-10395-0

**Published:** 2025-02-05

**Authors:** Regine Maier, Lukas Hörtnagl, Nina Buchmann

**Affiliations:** Institute of Agricultural Sciences, ETH Zurich, Universitätstrasse 2, 8092 Zurich, Switzerland

**Keywords:** Eddy covariance, Non-growing season, Greenhouse gas, Post-harvest, Crop residues

## Abstract

Global agriculture is the largest anthropogenic source for nitrous oxide (N_2_O) emissions. During crop rotations, periods with arable soils without crops, thereafter called “bare soils” are often impossible to avoid after the crop is harvested, prior to sowing of the next crop. However, such periods are underrepresented in studies focussing on N_2_O emissions. Here, we present continuous, high-temporal-resolution N_2_O fluxes during bare soil periods after four major crops, using the eddy-covariance technique at two sites in Switzerland. Overall, periods with bare soil were net sources for N_2_O as well as for carbon dioxide (CO_2_) and methane (CH_4_). Daily average sums of N_2_O emissions varied between 10 ± 2 and 38 ± 5 g N_2_O-N ha^−1^ d^−1^ after the respective rapeseed, winter wheat, pea, and maize harvests. While CO_2_ emissions contributed 86–96% to the total GHG budgets, N_2_O fluxes accounted for 2% after pea, but for 10–12% after rapeseed, winter wheat, and maize. In contrast, CH_4_ fluxes were negligible (< 2%). N_2_O fluxes during bare soil periods increased for all cropland sites with increasing water-filled pore space, particularly at high soil temperatures. Thus, our study emphasizes the significance of avoiding bare soil periods to mitigate N_2_O emissions from croplands.

**Supplementary Information:**

The online version contains supplementary material available at 10.1007/s10705-024-10395-0.

## Introduction

Nitrous oxide (N_2_O) is a potent long-lived greenhouse gas (GHG), the third most important anthropogenic GHG after carbon dioxide (CO_2_) and methane (CH_4_) (IPCC 2023). The largest anthropogenic source for N_2_O emissions worldwide is agriculture, with a total amount of 3.8 (2.5–5.8) Tg nitrogen (N) loss per year in the past decade, mainly due to N fertilizers applied to agricultural soils (Tian et al. 2020). With the increase of global food demand, N_2_O emissions are projected to further increase, raising further concerns about the future climate impacts of agriculture. Thus, understanding and quantifying N_2_O emissions are key for establishing N_2_O mitigation measures for croplands (Cui et al. 2024).

Nitrification and denitrification are the main microbial processes involved in N_2_O production in soils, both controlled by biotic, e.g., plant–microbe interactions, and abiotic drivers, e.g., soil N availability, soil water content or temperature (Butterbach-Bahl et al. 2013). Since these processes show very high spatial and temporal variabilities, continuous, high-resolution flux measurements are particularly well suited‒compared to chamber measurements—to capture their highly dynamic responses to changing conditions and to quantify total N_2_O-N losses, spatially integrated over entire fields (Feigenwinter et al. 2023a; Lognoul et al. 2019; Maier et al. 2022; Tallec et al. 2022). However, despite a wealth of chamber studies (e.g., Cui et al. 2021), which risk underestimating N_2_O emissions due to their limited spatial and temporal scale, only few studies exist using high temporal resolution (5–10 Hz) eddy-covariance measurements to investigate N_2_O fluxes from croplands. Moreover, often these studies focused only on the crop growing season, i.e., measuring from sowing to harvest of the respective crop (Huang et al. 2014; Lognoul et al. 2019; Maier et al. 2022; Tallec et al. 2019), while measurement campaigns are still urgently lacking that cover the non-growing seasons, i.e., the periods after crop harvest, prior to sowing the next crop, when arable soils are either bare or only covered by crop residues (Cui et al. 2021). These periods are sometimes called “post-harvest” or “open soil” periods, here called “bare soil” periods. This gap of knowledge is even more critical since such bare soil periods cannot be avoided in crop rotations (Shang et al. 2024), and differ substantially from true fallow periods, during which cropland is left idle for one entire growing season to restore soil fertility. While freeze–thaw cycles were reported to drive N_2_O emissions from croplands during the non-growing season in cold climates (Abalos et al. 2016; Quan et al. 2023; Wagner-Riddle et al. 2017), little information is available about N_2_O emissions during bare soil periods in temperate regions (Barton et al. 2011; Garland et al. 2014; Kennedy et al. 2013; Scheer et al. 2017; Walter et al. 2015). In addition, chamber study results during bare soil periods were ambiguous, reporting low as well as high N_2_O emissions after harvest, due to various reasons: (i) High temporal variations coinciding with precipitation or irrigation events, (ii) High spatial variations related to variable N availability from harvest residues, and (iii) N fertilization. Thus, bare soil contributions to total GHG budgets (i.e., CO_2_, N_2_O, and CH_4_) and emission factors for N_2_O during bare soil periods after harvests of different main crops remain elusive (Cui et al. 2021).

In this study, we therefore addressed the following objectives: (1) To quantify N_2_O emissions between harvest and sowing of four main crops, i.e., rapeseed, winter wheat, pea, and maize, at two different sites in Switzerland, (2) To identify the most important drivers for N_2_O losses, and (3) To assess GHG budgets for bare soil periods. We tested the following hypotheses: (1) N_2_O emissions are lower after the harvest of pea than after the harvest of rapeseed, winter wheat or maize, due to the match or mismatch of N supply vs. N demand during the respective preceding cropping seasons, (2) N_2_O fluxes during bare soil periods are more influenced by environmental drivers than by management activities, due to their strong control of microbial activities, and (3) Non-CO_2_ emissions do not dominate the respective GHG budgets, due to the presence of large soil carbon pools.

## Methods

### Site description

Measurements were performed at two sites, Oensingen and Aeschi, located on the Swiss Plateau, in the main agricultural area of the canton of Solothurn in Switzerland (Supplementary Table S1). Both sites experience continental temperate climate, with mean annual temperatures of around 9–10 °C and annual precipitation of about 1000–1100 mm (Emmel et al. 2018; Maier et al. 2022). The Oensingen site (OE2) is a long-term cropland site within the Swiss FluxNet and FLUXNET (CH-Oe2) where eddy covariance (EC) measurements of CO_2_ and H_2_O have been conducted since December 2003, making this site the second longest running cropland site globally (Emmel et al. 2018). The field is flat and uniform with an area of 1.55 ha. The soil is classified as Eutric-Stagnic Cambisol (FAO-WRB), with 43% clay, 47% silt, and 10% sand (Alaoui and Goetz 2008). Soil pH was 6.4, and bulk density was 1.24 Mg m^−3^ in the topsoil (0–0.1 m). The site has been managed under the Swiss integrated management regime since the 1990s, following a 3-year crop rotation with winter wheat as the main crop (Emmel et al. 2018).

The flux measurements at the Aeschi site (CH-AES) were running between 10 May 2019 and 3 November 2020, located approximately 13 km south of OE2 (Maier et al. 2022). The field had a size of 2.2 ha. The soil was classified as Gleyic Cambisol (FAO-WRB), with 23% clay, 41% silt, and 36% sand (loam). Soil pH in the topsoil (0–0.1 m) was 5.4, bulk density was 1.18 Mg m^−3^ (Supplementary Table S1). The field had been managed as a cropland for more than 20 years, with an alternating crop rotation between winter and summer crops such as winter cereal, winter rapeseed, maize, pea, and potato as well as clover-grass mixtures to restore soil health.

In this study, we focussed on the bare soil periods at both sites, i.e., periods after crop harvest and prior to sowing the next crop (for crop season fluxes at AES, see Maier et al. 2022). Thus, at OE2, we measured from 12 July 2018 until 11 October 2018 (after rapeseed; 92 days) and from 23 July 2019 (straw harvest) until 4 October 2019 (after winter wheat; 72 days). At AES, we measured from 3 July 2019 until 23 July 2019 (after pea; 20 days, missing the last 12 days of bare soil prior to sowing a grass-clover mixture), and from 16 September 2020 until 3 November 2020 (after silage maize; 48 days). During these bare soil periods, certain fertilizer applications or soil cultivation practices had been conducted by the farmers, representing typical Swiss management practices (Supplementary Table S2 for details on management activities at both sites).

### Carbon and nitrogen concentrations of crop residues

Crop residues were sampled immediately after all harvests at both sites, i.e., stubbles and harvest leftovers for rapeseed, winter wheat and maize, as well whole plants for pea (only the peas were harvested). A metal frame (0.1 m^2^) was used at four randomly selected locations within the footprint of the flux station. Samples were weighed and then dried at 60 °C for three days for moisture content calculation. From each sample, a small amount was milled (MM200, Retsch GmbH, Haan, Germany), and weighed into tin capsules for the determination of carbon (C) and N concentrations using a Flash EA 1112 series elemental analyser (Thermo Fisher Scientific—former CE Instruments, Rhodano, Italy). Mean C and N concentrations [%] of crop residues (n = 4) for each harvest were calculated and then scaled up to kg C or N dry weight ha^−1^ (Supplementary Table S2). C:N ratios of crop residues were calculated by dividing the amount of C in crop residues by the amount of N in crop residues (Supplementary Table S2), and compared well to earlier studies at those sites (Dietiker et al. 2010; Maier et al. 2022).

### Meteorological variables

The measurement setup at OE2 for meteorological variables used in this study consisted of a net radiometer (CNR1, Kipp & Zonen, Delft, Netherlands), a heated rain gauge (model 15,188, Lambrecht GmbH, Göttingen, Germany), and an air temperature and relative humidity sensor (CS215, Campbell Scientific Ltd., Logan UT, USA). Soil water content and soil temperature in the middle of the study site and its footprint were measured with one soil profile (at 0.05 m, 0.15 m, and 0.3 m soil depth; 5TM Water Content and Temperature Sensors, Decagon Devices, Inc., Pullman WA, USA). For details, see Emmel et al. (2018).

At AES, a weather station (ATMOS 41, METER Group, Inc. USA, Pullman WA, USA) was installed at the south-west corner of the field between 10 May 2019 and 3 November 2020. Solar radiation, precipitation, air temperature, and relative humidity were measured at 2 m height. For determination of soil water content and soil temperature, one soil profile with three soil sensors (5TM Water Content and Temperature Sensors, Decagon Devices, Inc., Pullman WA, USA) were installed at 0.05 m, 0.15 m, and 0.3 m soil depth.

All meteorological measurements were taken at 1-minute time resolution and after screening for outliers averaged to 30 min values. Water-filled pore space (WFPS; Eq. 1) was calculated as:

1 $$WFPS=\frac{SWC}{1-\frac{BD}{PD}}*100$$

where SWC represents soil water content at a specific depth (m^3^ m^−3^), BD is bulk density (1.24 Mg m^−3^ for OE2, and 1.18 Mg m^−3^ for AES), and PD represents particle density with 2.65 Mg m^−3^ (Danielson and Sutherland 1986).

### Eddy covariance measurements

The EC measurements were taken with an ultrasonic anemometer (R3-50, Gill Instruments Ltd., Lymington, UK) for three-dimensional wind speed and direction, an open-path infrared gas analyser (IRGA) for measurements of CO_2_ concentrations (LI-7500, LI-COR Biosciences, Lincoln NE, USA), and a laser spectrometer for measurements of N_2_O and CH_4_ concentrations (Rackmount—MIR, Enhanced Performance, Los Gatos Research, Mountain View, CA, USA). The latter was connected to an external pump (XDS35i, Edwards Ltd., Crawley, West Sussex, UK), both placed in temperature regulated boxes to maintain flow rate and cell pressure of the laser spectrometer at 12.5 L min ^−1^ and 45 Torr, respectively. A 7 m long and 7.5 mm wide tube (EATON Synflex 1300, Dublin, Ireland) equipped with an intake cap was used to draw air samples to the laser spectrometer. Filters placed right after the tube inlet were changed when clogged and cell pressure declined.

At OE2, the measurement height of the sonic anemometer and the IRGA was 2.17 m in 2018 (after winter rapeseed) and 2.26 m in 2019 (after winter wheat). At AES, the measurement height after pea was 2.18 m (2019) and after maize 2.62 m (2020). The inlet of the laser spectrometer was always mounted approximately 0.2 m below the sonic anemometer and the IRGA. Measurement rate of CO_2_ concentration was 20 Hz, while N_2_O and CH_4_ concentrations were recorded at 10 Hz. The whole EC system, i.e., ultrasonic anemometer, IRGA, laser spectrometer and the external pump, was moved between sites. At OE2, it was installed close to an existing EC system (Emmel et al. 2018), at AES it was placed in the centre of the field.

### Flux processing and flux quality control

The EC technique (Baldocchi 2003) was used to quantify turbulent fluxes of CO_2_, N_2_O, and CH_4_. Following established flux community guidelines (Aubinet et al. 2012; Pastorello et al. 2020), we used the software EddyPro (version 7.0.6, LI-COR Environmental, Lincoln NE, USA) to calculate 30 min averaged EC fluxes. Flux processing and flux quality control of raw EC data were performed for each year and each site separately. The raw EC data underwent despiking and screening, in line with Vickers and Mahrt's (1997) statistical tests. Wind data were rotated through 2D rotation (Wilczak et al. 2001), and time lags between turbulent departures of the vertical wind and scalar measurements (CO_2_ molar densities; N_2_O, CH_4_ mixing ratios) were determined by first identifying the absolute maximum of the cross-correlation function in a relatively large time window (0 to 10 s). Then, the frequency distribution of found time lags was used to identify typical lag ranges for each gas. In addition, the default (nominal) time lag was defined as the time lag corresponding to the peak of the distribution. Final time lags were calculated within a smaller time window (0 to 5 s). In case no clear covariance peak was found within this plausibility window, the default time lag was used instead. The molar density of CO_2_ accounted for ambient air density fluctuations (Webb et al. 1980). Spectral corrections addressed high-pass (Moncrieff et al. 2005) and low-pass filtering effects (CO_2_: Horst 1997; N_2_O and CH_4_: Fratini et al. 2012); in addition, instrument separation corrections for N_2_O and CH_4_ were applied (Horst and Lenschow 2009). The flux footprint model by Kljun et al. (2015) estimated the upwind area contributing to the measured GHG flux.

Flux quality control involved seven checks to ensure high-quality data for further analyses. Fluxes were discarded if (1) the steady-state test exceeded 30%, (2) the developed turbulent conditions test exceeded 30% (Foken et al. 2004), or (3) the spectral correction factor exceeded predefined values (< 2 = highest quality; between 2 and 4 = moderate quality, soft flag; > 4 = rejected, hard flag; Sabbatini et al. (2018). (4) CO_2_ fluxes were removed if the IRGA’s automatic gain control value indicated low CO_2_ signal quality (e.g., due to dirt on windows or in the optical path). Storage terms for CO_2_ were calculated according to Aubinet et al. (2001) and added to half-hourly CO_2_ flux to compute net ecosystem exchange (NEE). Storage terms for N_2_O and CH_4_ were neglected due to their assumed low values from low EC tower heights (Montagnani et al. 2018). All fluxes were (5) despiked by removing values outside the monthly mean ± 3 times its standard deviation (Rogiers et al. 2005). (6) Absolute limits were set to remove half-hourly fluxes outside physically plausible ranges (CO_2_: ± 50 μmol m^−2^ s^−1^; N_2_O: + 80 nmol m^−2^ s^−1^, − 50 nmol m^−2^ s^−1^; CH_4_: ± 500 nmol m^−2^ s^−1^). (7) During low friction velocity periods, a constant u⁎ threshold (Papale et al. 2006) was applied to NEE for both years (OE2 2018: 0.18 m s^−1^, OE2 2019: 0.19 m s^−1^; AES 2019: 0.16, AES 2020: 0.16 m s^−1^). The u⁎ threshold detection was performed using the R package *REddyProc* (Wutzler et al. 2018) and applied to N_2_O and CH_4_ fluxes as well.

In total, we directly measured 10,305 (NEE) and 10,611 (N_2_O and CH_4_) half-hourly EC fluxes across all bare soil periods and retained 61.9% (NEE), 73.6% (N_2_O) and 59.7% (CH_4_) with QC flags 0 (best quality fluxes) and 1 (medium quality fluxes). Low-quality fluxes (QC flag 2) were rejected from all analyses (Mauder and Foken 2004). The micrometeorological sign convention was used for reporting GHG fluxes, with negative values for uptake by the ecosystem and positive values for emissions to the atmosphere (Schulze et al. 2009).

### Gap-filling of GHG fluxes

Gap-filling of NEE was performed with the R package *REddyProc* (Wutzler et al. 2018) using marginal distribution sampling (Reichstein et al. 2005) (MDS), with three meteorological variables, i.e., global radiation (Rg), air temperature (TA), and vapor pressure deficit (VPD), with margins of ± 50 W m^−2^, ± 2.5 °C, and ± 5 hPa, respectively. The random forest (RF) algorithm (Breiman 2001) was used to gap-fill N_2_O and CH_4_ fluxes as it has been proven to be a valid method to gap-fill non-CO_2_ gases (Irvin et al. 2021; Kim et al. 2020). We used the R packages *randomForest* (Liaw and Wiener 2002) and *caret* (Kuhn 2022) with which we created bootstrapped datasets for each site, year and gas (N_2_O and CH_4_) separately. We then generated 1000 independent regression trees to predict missing half-hourly fluxes for N_2_O and CH_4_. The model was trained using only high-quality (QC flag 0) flux data. Furthermore, we estimated the “variable importance” to identify predictor variables for N_2_O and CH_4_. Details about predictor variables for N_2_O fluxes can be found in the Supplementary Table S3, and details on gap-filling and driver analysis can be found in Maier et al. (2022).

We used the RF model to calculate mean absolute errors (MAE) for N_2_O and CH_4_. In OE2, MAE were 0.34 nmol m^−2^ s^−1^ (2018) and 0.27 nmol m^−2^ s^−1^ (2019) for N_2_O fluxes, as well as 17.3 nmol m^−2^ s^−1^ (2018) and 9.4 nmol m^−2^ s^−1^ (2019) for CH_4_ fluxes. In AES, MAE were 0.2 nmol m^−2^ s^−1^ (2019) and 0.4 nmol m^−2^ s^−1^ (2019) for N_2_O fluxes, as well as 15.2 nmol m^−2^ s^−1^ (2019) and 12.9 nmol m^−2^ s^−1^ (2020) for CH_4_ fluxes.

### GHG budget calculation

For the GHG budget calculation of bare soil periods, we used gap-filled CO_2_ fluxes with MDS and gap-filled N_2_O and CH_4_ with RF. The total GHG budget does not include any further inputs or exports of C or N (in contrast to the NBP estimate, i.e., net biome production, which includes inputs and exports of C). Global warming potentials (GWP in CO_2_-equivalents) were calculated using current estimates of 273 for N_2_O and 27 for CH_4_ over a 100-year time horizon (IPCC 2023b).

## Results

### Meteorological conditions during bare soil periods

**Fig. 1:**
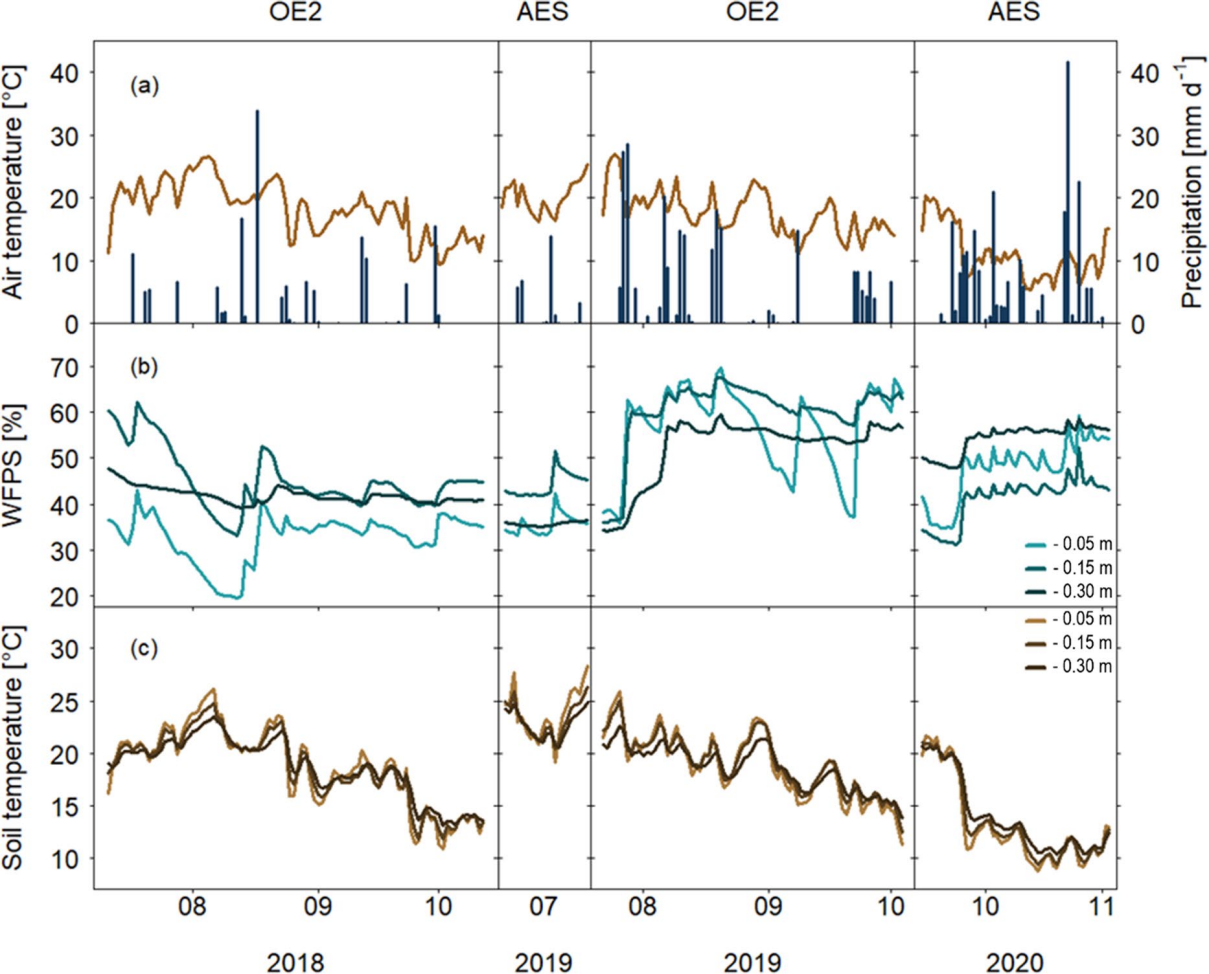
Environmental variables measured at Oensingen (OE2) and (AES) during periods with bare soils after harvests of rapeseed, pea, winter wheat, and maize (2018–2020). **a** Daily averaged air temperature [°C] and precipitation [mm d^−1^], **b** water-filled pore space in 0.05 m, 0.15 m and 0.3 m soil depths [WFPS, %], and **c** soil temperature [°C] in 0.05 m, 0.15 m and 0.3 m soil depths

Meteorological conditions differed between the two sites and investigated time periods. At OE2, the summer months in 2018 (after the rapeseed harvest) were affected by a severe drought, resulting in less precipitation (122 mm), lower water-filled pore space (WFPS; down to 18% over several days), but higher soil temperatures at all depths compared to those in summer 2019 (after the winter wheat harvest; Fig. 1). At AES, July 2019 (after pea harvest) was also very warm, with daily mean average air temperatures of 20.2 °C, and soil temperatures above 20 °C at all depths. In contrast, several days after the maize harvest in mid-September 2020 (AES), a cold front with intense rainfall resulted in a drop of air and soil temperatures and an increase of WFPS at all soil depths (Fig. 1).

### Nitrous oxide fluxes during bare soil periods

**Fig. 2:**
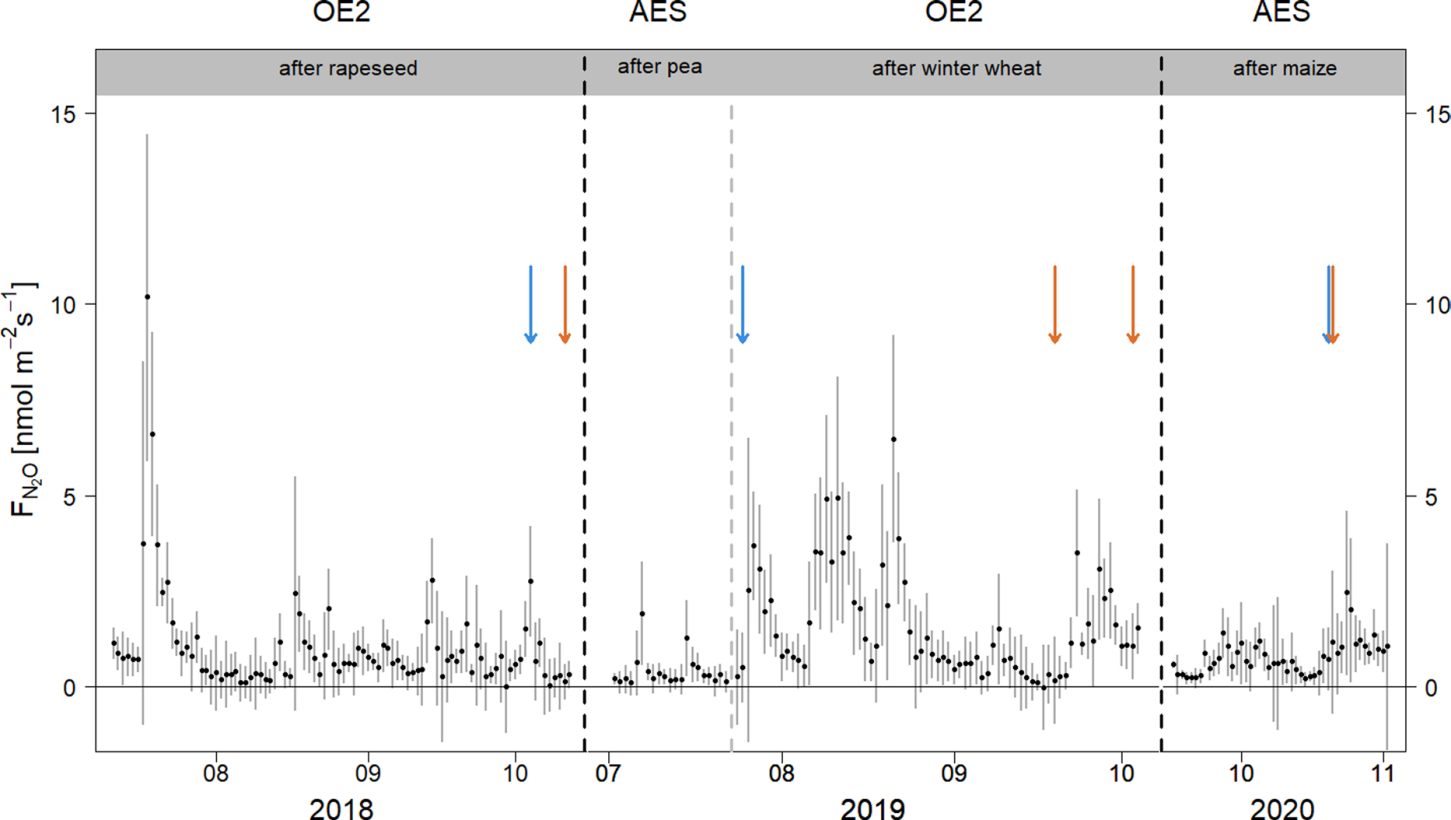
Daily averaged N_2_O fluxes [nmol m^−2^ s^−1^, black dots] at Oensingen (OE2) and Aeschi (AES) during periods with bare soils in 2018 to 2020 after the harvests of rapeseed, pea, winter wheat, and maize, before sowing the next crop. Dashed lines (black and grey) indicate the end of the respective measurement period at any given site. In addition, black dashed lines show a break in the time axis. Blue arrows indicate N fertilizer applications, and orange arrows indicate soil cultivation. Means ± standard deviations are given. See Supplementary Table S2 for details on management activities

In this study, we quantified N_2_O fluxes from arable soils after the respective harvest of four major crops in Switzerland, i.e., winter rapeseed, winter wheat, pea, and maize. We measured N_2_O fluxes after rapeseed (92 days in 2018) and after winter wheat (72 days in 2019) at OE2 as well as after pea (20 days in 2019) and after silage maize (48 days in 2020) at AES. During these periods, both croplands were clear N_2_O sources, with considerable short-term variations and higher daily averaged N_2_O fluxes after rapeseed and winter wheat compared to those after pea and maize (Fig. 2). Averaged and summed up over a day, N_2_O fluxes were lowest after pea (10 ± 2 g N ha^−1^), moderate after maize (19 ± 3 g N ha^−1^), and highest after rapeseed (25 ± 4 g N ha^−1^) and winter wheat (38 ± 5 g N ha^−1^, Table 1). This results in cumulative N_2_O losses of 2.8 ± 0.4 kg N ha^−1^ over 72 days after winter wheat, 2.3 ± 0.4 kg N ha^−1^ over 92 days after rapeseed, 0.9 ± 0.2 kg N ha^−1^ over 48 days after maize, and 0.2 ± 0.05 kg N ha^−1^ over 20 days after pea (Table 1). The uncertainty of cumulative sums for N_2_O emissions as calculated in Table 1 lies between 14 and 22% which is in the range reported by Lognoul et al. (2019) with 0.3 kg N ha^−1^ for a cropping season of beet root.

**Table 1:** Daily average sums ± standard deviation and cumulative sums ± standard deviation of N_2_O, CO_2_, and CH_4_ fluxes [in g or kg N ha^−1^ and g or kg C ha^−1^] for Oensingen (OE2) and Aeschi (AES) during periods with bare soils in 2018 to 2020 after the harvests of rapeseed, winter wheat, pea, and maize prior to sowing the next crop

Site	Flux	N_2_O	CO_2_	CH_4_
Oensingen (OE2)				
After rapeseed	Daily average sum	25 ± 4 g N ha^−1^	29 ± 2 kg C ha^−1^	48 ± 5 g C ha^−1^
	Cumulative sum [92 days]	2.3 ± 0.4 kg N ha^−1^	2645 ± 220 kg C ha^−1^	4.4 ± 0.5 kg C ha^−1^
After winter wheat	Daily average sum	38 ± 5 g N ha^−1^	38 ± 6 kg C ha^−1^	11 ± 2 g C ha^−1^
	Cumulative sum [72 days]	2.8 ± 0.4 kg N ha^−1^	2744 ± 397 kg C ha^−1^	0.8 ± 0.2 kg C ha^−1^
Aeschi (AES)				
After pea	Daily average sum	10 ± 2 g N ha^−1^	46 ± 6 kg C ha^−1^	18 ± 4 g C ha^−1^
	Cumulative sum [20 days]	0.2 ± 0.05 kg N ha^−1^	918 ± 117 kg C ha^−1^	0.4 ± 0.08 kg C ha^−1^
After maize	Daily average sum	19 ± 3 g N ha^−1^	17 ± 3 kg C ha^−1^	28 ± 3 g C ha^−1^
	Cumulative sum [48 days]	0.9 ± 0.2 kg N ha^−1^	815 ± 160 kg C ha^−1^	1.3 ± 0.2 kg C ha^−1^

Positive values indicate emissions to the atmosphere

### Drivers for N_2_O fluxes

**Fig. 3:**
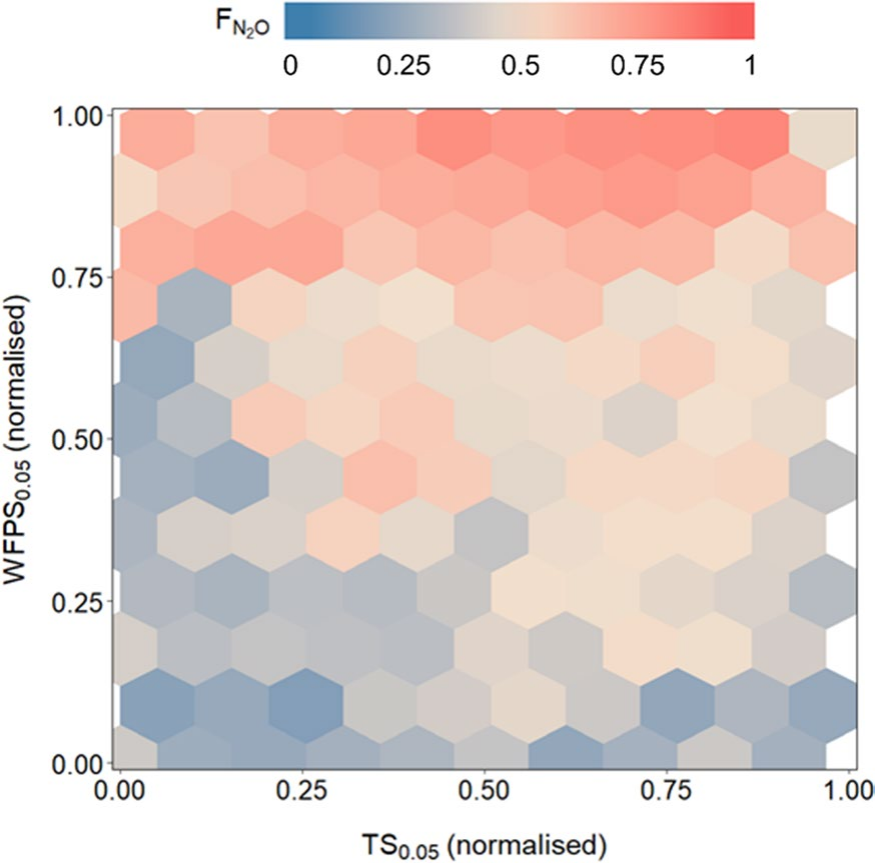
Hexbin plots with normalised soil temperature and normalised water-filled pore space at 0.05 m soil depth and non-gap filled normalised half-hourly N_2_O fluxes for both sites (OE2 and AES) during periods with bare soils (2018–2020). Only high-quality flux data (QCF 0) were used. Normalisation was performed using the empirical cumulative distribution function for each year and site separately

Our analysis for N_2_O fluxes with random forest (RF) showed that during all investigated bare soil periods at croplands, typically WFPS and soil temperature (TS) at 0.05 m depth had the highest importance among all input variables (variable importance with RF model: WFPS 17.1–30, TS 12.9–22.2; Supplement Fig. S3). The magnitude of bare soil N_2_O fluxes increased for both our cropland sites with increasing WFPS, particularly at high soil temperatures (Fig. 3). Such favourable conditions for soil N dynamics were, for example, observed after the winter wheat harvest in fall 2019, resulting in the highest sum of daily N_2_O fluxes in this study (38 ± 5 g N_2_O-N ha^−1^ d^−1^; Table 1). In contrast, N_2_O fluxes declined with decreasing soil temperatures (after maize harvest in 2020) or with low WFPS (about 35% WFPS after pea in summer 2019), resulting in small N_2_O emissions. Thus, WFPS and soil temperature were the most important drivers for N_2_O fluxes from arable soils during periods with bare soils, independent of crop type.

### Contributions to the total GHG budget

The total GHG budgets of the four bare soil periods were all positive, i.e., bare soils were a net source for N_2_O, CO_2_ and CH_4_, ranging from 342 to 1128 g CO_2_-eq m^−2^ across both sites for the respective measurement periods (Table 2). The contribution of N_2_O fluxes to the respective total GHG budgets was between 10 and 12% for bare soil periods after the harvests of rapeseed, winter wheat, and maize, while their contribution after pea was considerably lower (2%; Table 2). As expected, CO_2_ fluxes during these periods dominated the GHG flux, contributing 86–96% to the total GHG budget. Highest daily average CO_2_ losses were measured after pea (46 ± 6 kg CO_2_-C ha^−1^ d^−1^), while lowest CO_2_ losses were measured after maize (17 ± 3 kg CO_2_-C ha^−1^ d^−1^; Table 1; Supplementary Fig. S1). CH_4_ fluxes during periods with bare soil were negligible (11 ± 2–48 ± 5 g CH_4_-C ha^−1^ d^−1^; Table 1; Supplementary Fig. S2), only contributing between 0.3 and 2% to the total GHG budget.

**Table 2:** CO_2_-equivalents [g CO_2_-eq m^−2^] calculated for N_2_O, CO_2_, and CH_4_ fluxes for Oensingen (OE2) and Aeschi (AES) during periods with bare soil after harvests of rapeseed, winter wheat, pea, and maize, before sowing the next crop

Site	Flux	N_2_O	CO_2_	CH_4_	Sum
Oensingen (OE2)					
After rapeseed	CO_2_-eq per day	1.1	10.5	0.2	+ 12
	CO_2_-eq cumulative [92 days]	98	969	16	+ 1083
After winter wheat	CO_2_-eq per day	1.7	13.9	0.04	+ 16
	CO_2_-eq cumulative [72 days]	119	1006	3	+ 1128
Aeschi (AES)					
After pea	CO_2_-eq per day	0.4	16.8	0.05	+ 17
	CO_2_-eq cumulative [20 days]	9	337	1	+ 347
After maize	CO_2_-eq per day	0.8	6.2	0.1	+ 7
	CO_2_-eq cumulative [48 days]	38	299	5	+ 342

Positive values indicate emissions to the atmosphere. CO_2_-equivalents were calculated by multiplying cumulative sums of N_2_O and CH_4_ fluxes over the measurement period by their global warming potentials of 273 and 27, respectively

## Discussion

### Nitrous oxide fluxes during bare soil periods

The highest daily N_2_O emission peaks were observed after the harvest of rapeseed at OE2, intermediate after the harvest of winter wheat and maize, and smallest after pea harvest (Fig. 2). However, daily average sums of N_2_O emissions were largest during the bare soil period after the harvest of winter wheat, followed by that of rapeseed and maize, but lowest after the harvest of pea (Tables 1 and 2). Low N_2_O emissions at AES after both pea and maize harvests could be explained by environmental conditions suppressing microbial activities for denitrification (Butterbach-Bahl et al. 2013; Cowan et al. 2020). In July 2019 after pea harvest, dry and warm conditions (Fig. 1) while after maize harvest in mid-September 2020 with wet conditions but low soil temperatures, N_2_O emissions were also rather low. Despite the dry conditions at OE2 during summer 2018, we measured high N_2_O emissions immediately after rapeseed harvest (Table 1). The N_2_O peak fluxes after harvest might have been triggered by the high N inputs after the harvest via crop residues of rapeseed (97 kg N ha^−1^; Supplementary Table S2) with a low C:N ratio of 34 ± 5 (Supplementary Table S2, since N inputs via manure application towards the end of the bare soil period were rather small (5 kg N ha^−1^). It has been shown that C:N ratios of crop residues < 45 provide easily available N for soil microbes, stimulating nitrification and denitrification and thus N_2_O emissions as observed during the bare soil period after rapeseed (Abalos et al. 2022b; Chen et al. 2013). In contrast, peak N_2_O emissions after winter wheat and maize were smaller than that after rapeseed, despite a compost application at the beginning of the bare soil period after the winter wheat harvest, contributing to the highest average daily N_2_O fluxes. C:N ratios > 45, as found for winter wheat straw and maize (83 ± 15 and 77 ± 8, respectively; Supplementary Table S2), together with the very low N inputs via crop residues of winter wheat (2 kg N ha^−1^), but medium N inputs via compost applications (22 kg N ha^−1^; Supplementary Table S2), could have resulted in lower peak N_2_O emissions immediately after winter wheat harvest compared to rapeseed harvest, maybe due to net N immobilization (Abalos et al. 2022a; Chen et al. 2013). In contrast, after the pea harvest, N inputs from crop residues were intermediate (46 kg N ha^−1^) with a low C:N ratio of 34 ± 4, but peak N_2_O emissions during the bare soil period remained low (Fig. 2, Table 1). After maize harvest, N inputs via crop residues (albeit with a very wide range of C:N ratios) and slurry application were relatively high (33 and 54 kg N ha^−1^, respectively); however, peak N_2_O emissions after maize were lower compared to fluxes after rapeseed and winter wheat, implying that C:N ratios of crop residues and N inputs are clearly not the sole driver for N_2_O emissions (Abalos et al. 2022b).

Our driver analysis (Supplementary Fig. S3) showed that for both sites, WFPS and soil temperature were crucial environmental factors to predict N_2_O fluxes, as generally reported for croplands and grasslands (Butterbach-Bahl et al. 2013; Liang et al. 2018; Hörtnagl et al. 2018; Lognoul et al. 2019). These results supported the notion that any mismatch of soil N supply to plant N demand—fully lacking during bare soil periods–resulted in considerable, although avoidable N_2_O fluxes, which can reach up to 2.8 ± 0.4 kg N ha^−1^ within 2.5 months.

### Contribution to the GHG budget

Total GHG budgets from croplands are rare due to the lack of high-resolution studies investigating all three GHGs (Quan et al. 2023). This lack of cropland sites became evident in a global flux data compilation for net ecosystem CO_2_ exchange measurements (FLUXNET2015; Pastorello et al. 2020). There, only 20 cropland and 37 grassland sites could be included, accounting for only about 10% and 18%, respectively, of the ‒in total‒ 212 active flux sites, compared to 100 forest sites (47%). High-resolution measurements of non-CO_2_ gas fluxes (i.e., CH_4_ and N_2_O) are even less wide-spread, particularly for croplands, triggering “Research priority 1: Extending N_2_O observations and manipulation experiments” in a global review on N_2_O mitigation options by Cui et al. (2024). With the clear lack of N_2_O flux data during bare soil periods, i.e., after any cropping season but before sowing the next crop, our study clearly fills a gap of knowledge. For our two sites, the contributions of CH_4_ to the total GHG budget were negligible (0.3 and 2%) compared to CO_2_, which was clearly the dominant GHG flux across both sites and all crops, with 86–96% contributions. However, N_2_O emissions measured during bare soil periods amounted to 10–12% in our study (except for pea), remaining below the range of N_2_O contributions reported for bare soils elsewhere. For example, Quan et al. (2023) found that between 61 and 90% of the annual N_2_O emissions occurred during the non-growing season between a potato and a pea growing season in Western Canada. As the non-growing season in Canada was in winter, i.e., between mid-September and mid-June of the following year, it was characterized by heavy precipitation. Therefore, contributions of GHG fluxes during bare soil periods will vary with location and management, increasing uncertainties for cropland models as well as for mitigation strategies.

Moreover, in a companion study, Maier et al. (2022) reported that the total GHG budgets for the respective growing seasons of pea and maize at AES were GHG sinks for both crops (− 453 g CO_2_-eq m^−2^ for pea; − 1733 g CO_2_-eq m^−2^ for maize). Thus, the GHG sink of the pea crop would drastically change when the period with bare soil were also to be considered (with + 347 g CO_2_-eq m^−2^), i.e., if the one month after the harvest were included, resulting in a GHG sink of only − 106 g CO_2_-eq m^−2^. For maize, the consequences of including the bare soil period would be smaller than for pea, resulting in a GHG sink of still − 1391 g CO_2_-eq m^−2^. Hence, in order to reliably estimate the GHG emissions and thus the climate impact of crop rotations, periods with bare soils must also be considered in measurement campaigns (e.g., Butterbach-Bahl et al. 2013; Quan et al. 2023; Wagner-Riddle et al. 2020). If these bare soil periods were neglected, total N_2_O emissions and thus GHG budgets of croplands will be clearly underestimated. This perspective is supported by Shang et al. (2024), who emphasized the significant role of fallow period N_2_O emissions, which accounted for approximately 44% of the annual total N_2_O emissions globally, suggesting a critical assessment of emission factors for N_2_O in national greenhouse gas inventories.

### Future implications of N_2_O fluxes for agricultural practices

It has recently been shown that vegetation plays an important role in the N_2_O exchange during the growing season of both cropland and grassland (Feigenwinter et al. 2023a; Maier et al. 2022; Tallec et al. 2022; Timilsina et al. 2024), even beyond N fertilization and soil conditions (e.g., temperature, WFPS). Such results are not surprising since any surplus soil nitrate availability, the substrate for nitrification and denitrification, results from a mismatch between plant N uptake and N supply, pointing to a strong competition between soil microorganisms and plants, independent of soil conditions. Low plant N uptake early in the year (e.g., during periods with still cold, maybe wet soils) or right after sowing anytime during the year (e.g., during warm summer conditions) combined with large N supply via N fertilization or mineralization strongly increase the risk for N_2_O emissions as well as nitrate leaching (e.g., Butterbach-Bahl et al. 2013; Cui et al. 2024; Tenuta et al. 2019), most likely since small plants with their still small root systems lose the competition for N against soil microorganisms. When plant N uptake increases, even under wet soil conditions, N_2_O emissions and nitrate leaching are less likely to occur, demonstrated in site-specific studies in croplands (Maier et al. 2022; Tallec et al. 2022) and grasslands (Feigenwinter et al. 2023a, b). At the global level, Cui et al. (2024) concluded that physiological and ecological characteristics of plants belong to the underlying mechanisms to reduce N losses as N_2_O or nitrate, pointing to a wide range of potential agricultural practices beyond N fertilization, e.g., timing of management practices, crop selection, mixtures, intercropping to name a few. Thus, plant-adapted N fertilization has the potential to mitigate N_2_O emissions when vegetation is present. How precision farming, spatially and temporally adapted to the specific cropland, can play a major role, remains to be seen, particularly in terms of economic sustainability (Finger et al. 2019; Walter et al. 2017).

Moreover, reducing N fertilization alone can reduce N_2_O emissions (Feigenwinter et al. 2023a; Fuchs et al. 2018), however, it might also jeopardize the soil C sink, particularly under organic manure and/or slurry fertilization (Feigenwinter et al. 2023b). Solving this trade-off between reducing N_2_O emissions while maintaining the soil C sink, e.g., with nitrification inhibitors (Ruser and Schulz 2015), poses still a great challenge. Nevertheless, avoiding long periods with bare soil, e.g., with the use of cover crops, can potentially be an important mitigation step to reduce N_2_O emissions from croplands (Basche et al. 2014; Wang et al. 2021). Growing cover crops has further benefits, e.g., reduced nitrate leaching (Abdalla et al. 2019), improved soil health, or even increased crop yields (Garland et al. 2021). Modelling CO_2_ fluxes for cover crops vs. bare soil for OE2 clearly showed that cover crops reduced CO_2_ losses during periods with bare soil from this rather productive site (Emmel et al. 2018). Although cover crops have shown promise in mitigating CO_2_ emissions during bare soil periods at OE2, their impact on N_2_O emissions remains uncertain. Additional measurements or modelling studies are necessary to verify this relationship and to assess the overall environmental benefits of cover crop adoption. Finally, in order to develop best agricultural N_2_O mitigation strategies for agricultural management of croplands, accounting for the full crop rotation is key, and thus will require an increasing number of high-resolution measurements of N_2_O fluxes. Recent efforts of developing a *Global Database for N*_*2*_*O* (Dorich et al. 2020) will support solving this urgent need, providing N_2_O data following the FAIR principles (**F**indability, **A**ccessibility, **I**nteroperability, and **R**euse of data).

## Conclusions

The presented study provided high-temporal-resolution N_2_O flux data during the non-growing season, i.e., after crop harvest and before sowing of the next crop, advancing our understanding of the exchange of N_2_O from arable soils. Thus, closing this knowledge gap about the magnitude of N_2_O emissions during bare soil periods within crop rotations seems crucial for the transformation towards more sustainable agriculture, since these periods occur regularly, but might be shortened, if they cannot be fully avoided. How relevant N_2_O emissions during bare soil periods are under different environmental conditions and for other crop species needs to be shown globally. However, full cropping season measurements of N_2_O fluxes will clearly help to identify the legacy effects of any match or mismatch of N supply vs. plant N demand and help to develop suitable N_2_O mitigation practices without compromising soil C sequestration.

## Supplementary Information

Below is the link to the electronic supplementary material.

Supplementary file1 (DOCX 706 KB)

## Data Availability

The datasets generated and analysed during this study are openly available from the ETH Zurich Research Collection (10.3929/ethz-b-000584890).
