# Supplementary material for: Large nitrous oxide emissions from arable soils after crop harvests prior to sowing
Source: Nutr Cycl Agroecosyst. 2025 Feb 5;130(2):161–75. doi: 10.1007/s10705-024-10395-0 (PMC11950146; doi:10.1007/s10705-024-10395-0)
Supplement: Supplementary file 1 — Supplementary file1 (DOCX 706 KB) [file 10705_2024_10395_MOESM1_ESM.docx]

# Supplementary material for manuscript

# “Large nitrous oxide emissions from arable soils after crop harvests prior to sowing”

Regine Maier^1*^, Lukas Hörtnagl^1^ and Nina Buchmann^1^

^1^ Department of Environmental Systems Science, Institute of Agricultural Sciences, ETH Zurich, Universitätstrasse 2, 8092 Zurich, Switzerland

^*^Corresponding author: Regine Maier, regine.maier@wsl.ch


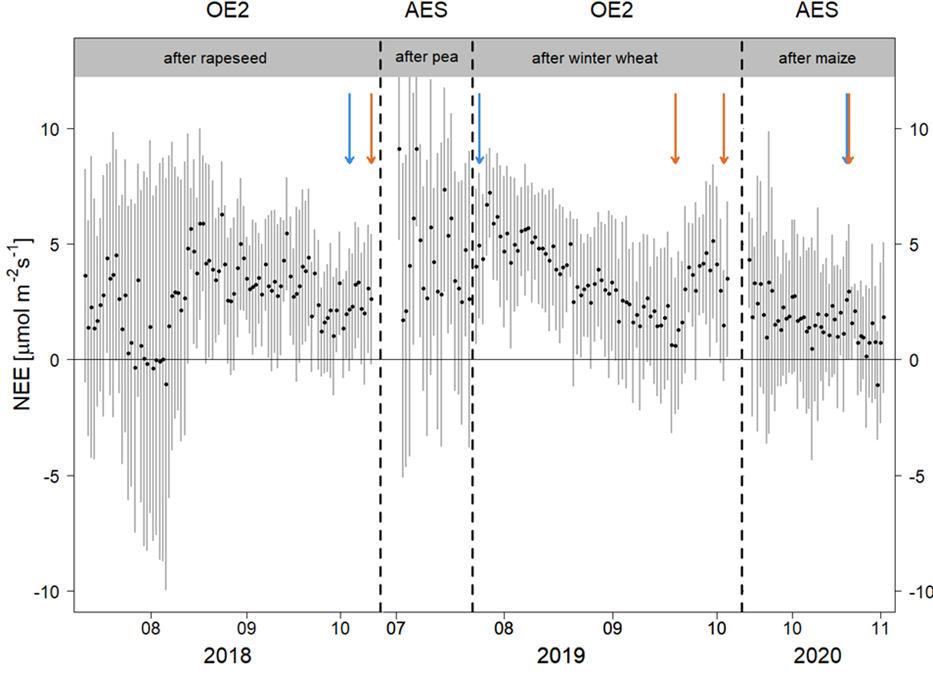


Figure S 1: Daily averaged net ecosystem CO_2_ exchange [NEE; black dots, µmol m^-2^ s^-1^] at Oensingen (OE2) and Aeschi (AES) during periods with bare soils after harvests of rapeseed, pea, winter wheat, and maize. Dashed lines indicate the end of the respective measurement period at any given site, due to the rotation of the instrument. Blue arrows indicate fertilizer applications, and orange arrows indicate soil cultivation. Means ± standard deviations are given. See Supplementary Tab. S 2 for details on management activities.


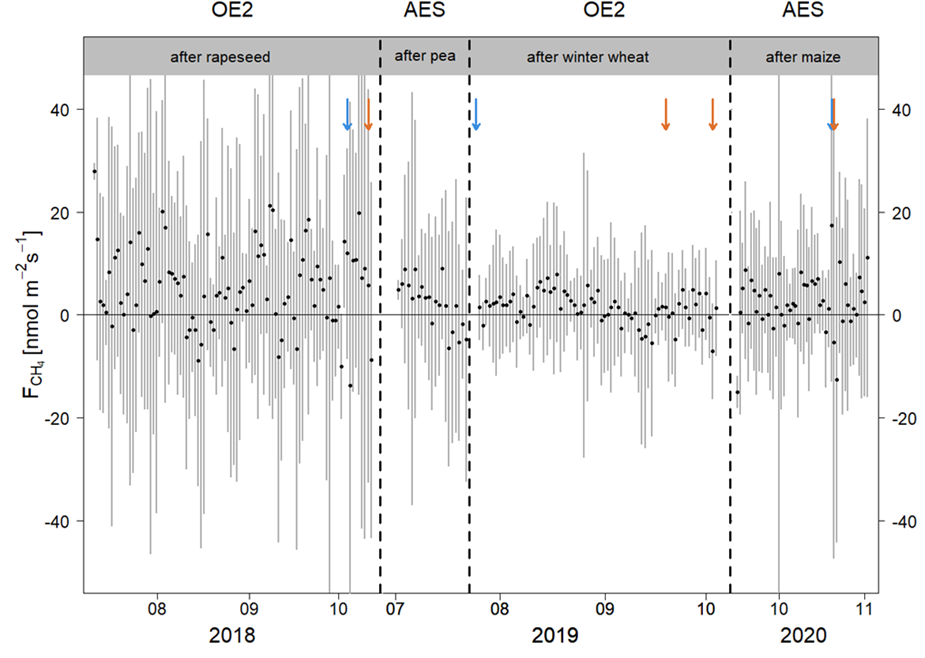


Figure S 2: Daily averaged CH_4_ fluxes [black dots, nmol m^-2^ s^-1^] at Oensingen (OE2) and Aeschi (AES) during periods with bare soils after harvests of rapeseed, pea, winter wheat, and maize. Dashed lines indicate the end of the respective measurement period at any given site, due to the rotation of the instrument. Blue arrows indicate fertilizer applications, and orange arrows indicate soil cultivation. Means ± standard deviations are given. See Supplementary Tab. S 2 for details on management activities.


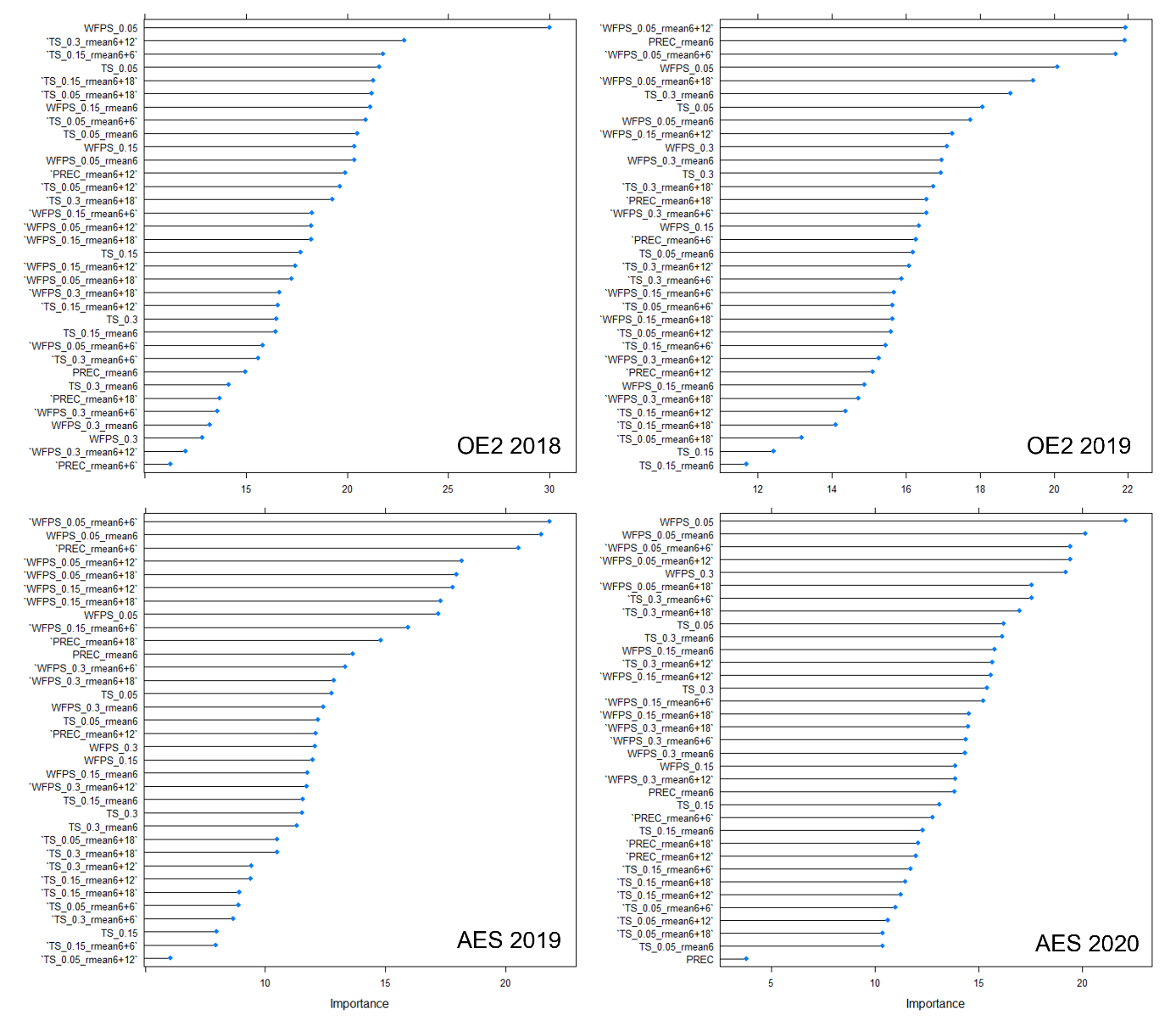


Figure S 3: Driver analyses for N_2_O fluxes using random forest. The 20 most important drivers are shown for each site (OE2 and AES) and year (2018-2020) separately. The line length indicates the feature importance of each variable to predict half-hourly N_2_O fluxes. For details and abbreviations, see Methods in the main text.

Table S 1: Description of both study sites, Oensingen (OE2) and Aeschi (AES)

| Site | Oensingen (OE2) | Aeschi (AES) |
| --- | --- | --- |
| Coordinates | 47.28 N, 7.73 E | 47.17 N, 7.66 E |
| Elevation [m a.s.l] | 452 | 465 |
| Area [ha] | 1.55 | 2.2 |
| Soil texture | silty clay | loam |
| pH^§^ | 6.4 | 5.4 |
| Bulk density [Mg m^-3^]^§^ | 1.24 | 1.18 |
| Measurement period | 12 July 2018 – 11 October 2018  (92 days with bare soil; rapeseed)  23 July 2019 – 4 October 2019  (72 days with bare soil; winter wheat) | 4 July 2019 – 23 July 2019  (20 days with bare soil; pea)  16 September 2020 – 3 November 2020 (48 days with bare soil; maize) |

^§^ pH and bulk density from topsoil 0-0.1 m

Table S 2: Management activities including biomass, C and N inputs during periods of bare soil after harvests of rapeseed, winter wheat, pea, and maize at Oensingen (OE2) and Aeschi (AES) between 2018 and 2020. Negative values indicate inputs to the field [kg C or N ha^-1^].

| Site | Bare soil | Management | Date | Amount | | | C:N ratio |
| --- | --- | --- | --- | --- | --- | --- | --- |
|  |  |  |  | kg ha^-1^ | kg C ha^-1^ | kg N ha^-1^ |  |
| OE2 | After rapeseed | Crop residues after harvest | 12 July 2018 | - | -3316 | -97 | 34 ± 5 |
|  |  | Solid manure application | 4 October 2018 | -10’000 | -224 | -5 | 45 |
|  |  | Mineral fertilizer application (PK) | 9 October 2018 | -200 | - | - |  |
|  |  | Soil cultivation | 11 October 2018 | - | - | - |  |
|  |  | Sowing winter wheat | 11 October 2018 | -180 | -69 | -3 | 23 |
|  | After winter wheat | Crop residues after harvest | 23 July 2019 | - | -152 | -1.8 | 83 ± 15 |
|  |  | Compost application | 25 July 2019 | -50 m^-3^ | -725 | -22 | 33 |
|  |  | Harrowing | 19 September 2019 | - | - | - |  |
|  |  | Soil cultivation | 3 October 2019 | - | - | - |  |
|  |  | Sowing winter barley | 4 October 2019 | -150 | -57 | -3 | 19 |
| AES | After pea | Crop residues after harvest^§^ | 3 July 2019 | - | -1517 | -46 | 34 ± 4 |
|  | After maize | Crop residues after harvest | 16 September 2020 | - | -2824 | -37 | 77 ± 8 |
|  |  | Slurry application | 20 October 2020 | -30 m^3^ | -266 | -54 | 5 |
|  |  | Soil cultivation | 21 October 2020 | - | - | - |  |

^§^Note: Only the peas were harvested, thus crop residues consisted of whole pea plants including pods.

Table S3: Predictor variables for RF models for gap-filling and driver analysis of N_2_O fluxes and used for each site and year separately.

| **Measurement** | **Variable name** | **Description** |
| --- | --- | --- |
| Air temperature | TA | Air temperature at 2 m height (°C) |
| Incoming short-wave radiation | Rg | Incoming short-wave radiation (W m^-2^) |
| Precipitation | PREC | precipitation (mm) |
| Water-filled pore space | WFPS_0.05 | WFPS in 5 cm depth (%) |
| Water-filled pore space | WFPS_0.15 | WFPS in 15 cm depth (%) |
| Water-filled pore space | WFPS_0.30 | WFPS in 30 cm depth (%) |
| Soil temperature | TS_0.05 | TS in 5 cm depth (°C) |
| Soil temperature | TS_0.15 | TS in 15 cm depth (°C) |
| Soil temperature | TS_0.30 | TS in 30 cm depth (°C) |
| Air temperature | TA_rmean6 | TA, 3-hour mean over preceding 3 hours |
| Water-filled pore space | WFPS_0.05_rmean6 | WFPS in 5 cm depth, 3-hour mean over preceding 3 hours |
| Water-filled pore space | WFPS_0.15_rmean6 | WFPS in 15 cm depth, 3-hour mean over preceding 3 hours |
| Water-filled pore space | WFPS_0.3_rmean6 | WFPS in 30 cm depth, 3-hour mean over preceding 3 hours |
| Soil temperature | TS_0.05_rmean6 | TS in 5 cm depth, 3-hour mean over preceding 3 hours |
| Soil temperature | TS_0.15_rmean6 | TS in 15 cm depth, 3-hour mean over preceding 3 hours |
| Soil temperature | TS_0.3_rmean6 | TS in 30 cm depth, 3-hour mean over preceding 3 hours |
| Water-filled pore space | WFPS_0.05.r-mean6+6 | WFPS in 5 cm depth (%), 3-hour mean ending 3 hours ago |
| Water-filled pore space | WFPS_0.05.r-mean6+12 | WFPS in 5 cm depth (%), 3-hour mean ending 6 hours ago |
| Water-filled pore space | WFPS_0.05.r-mean6+18 | WFPS in 5 cm depth (%), 3-hour mean ending 9 hours ago |
| Water-filled pore space | WFPS_0.15.r-mean6+6 | WFPS in 15 cm depth (%), 3-hour mean ending 3 hours ago |
| Water-filled pore space | WFPS_0.15.r-mean6+12 | WFPS in 15 cm depth (%), 3-hour mean ending 6 hours ago |
| Water-filled pore space | WFPS_0.15.r-mean6+18 | WFPS in 15 cm depth (%), 3-hour mean ending 9 hours ago |
| Water-filled pore space | WFPS_0.30.r-mean6+6 | WFPS in 30 cm depth (%), 3-hour mean ending 3 hours ago |
| Water-filled pore space | WFPS_0.30.r-mean6+12 | WFPS in 30 cm depth (%), 3-hour mean ending 6 hours ago |
| Water-filled pore space | WFPS_0.30.r-mean6+18 | WFPS in 30 cm depth (%), 3-hour mean ending 9 hours ago |
| Soil temperature | TS_0.05.r-mean6+6 | TS in 5 cm depth (°C), 3-hour mean ending 3 hours ago |
| Soil temperature | TS_0.05.r-mean6+12 | TS in 5 cm depth (°C), 3-hour mean ending 6 hours ago |
| Soil temperature | TS_0.05.r-mean6+18 | TS in 5 cm depth (°C), 3-hour mean ending 9 hours ago |
| Soil temperature | TS_0.15.r-mean6+6 | TS in 15 cm depth (°C), 3-hour mean ending 3 hours ago |
| Soil temperature | TS_0.15.r-mean6+12 | TS in 15 cm depth (°C), 3-hour mean ending 6 hours ago |
| Soil temperature | TS_0.15.r-mean6+18 | TS in 15 cm depth (°C), 3-hour mean ending 9 hours ago |
| Soil temperature | TS_0.30.r-mean6+6 | TS in 30 cm depth (°C), 3-hour mean ending 3 hours ago |
| Soil temperature | TS_0.30.r-mean6+12 | TS in 30 cm depth (°C), 3-hour mean ending 6 hours ago |
| Soil temperature | TS_0.30.r-mean6+18 | TS in 30 cm depth (°C), 3-hour mean ending 9 hours ago |
| Precipitation | PREC_r-mean6 | precipitation (mm), 3-hour mean over preceding 3 hours |
| Precipitation | PREC_r-mean6+6 | precipitation (mm), 3-hour mean ending 3 hours ago |
| Precipitation | PREC_r-mean6+12 | precipitation (mm), 3-hour mean ending 6 hours ago |
| Precipitation | PREC_r-mean6+18 | precipitation (mm), 3-hour mean ending 9 hours ago |

| **Measurement** | **Variable name** | **Description** |
| --- | --- | --- |
| Air temperature | TA | Air temperature at 2 m height (°C) |
| Incoming short-wave radiation | Rg | Incoming short-wave radiation (W m^-2^) |
| Precipitation | PREC | precipitation (mm) |
| Water-filled pore space | WFPS_0.05 | WFPS in 5 cm depth (%) |
| Water-filled pore space | WFPS_0.15 | WFPS in 15 cm depth (%) |
| Water-filled pore space | WFPS_0.30 | WFPS in 30 cm depth (%) |
| Soil temperature | TS_0.05 | TS in 5 cm depth (°C) |
| Soil temperature | TS_0.15 | TS in 15 cm depth (°C) |
| Soil temperature | TS_0.30 | TS in 30 cm depth (°C) |
| Air temperature | TA_rmean6 | TA, 3-hour mean over preceding 3 hours |
| Water-filled pore space | WFPS_0.05_rmean6 | WFPS in 5 cm depth, 3-hour mean over preceding 3 hours |
| Water-filled pore space | WFPS_0.15_rmean6 | WFPS in 15 cm depth, 3-hour mean over preceding 3 hours |
| Water-filled pore space | WFPS_0.3_rmean6 | WFPS in 30 cm depth, 3-hour mean over preceding 3 hours |
| Soil temperature | TS_0.05_rmean6 | TS in 5 cm depth, 3-hour mean over preceding 3 hours |
| Soil temperature | TS_0.15_rmean6 | TS in 15 cm depth, 3-hour mean over preceding 3 hours |
| Soil temperature | TS_0.3_rmean6 | TS in 30 cm depth, 3-hour mean over preceding 3 hours |
| Water-filled pore space | WFPS_0.05.r-mean6+6 | WFPS in 5 cm depth (%), 3-hour mean ending 3 hours ago |
| Water-filled pore space | WFPS_0.05.r-mean6+12 | WFPS in 5 cm depth (%), 3-hour mean ending 6 hours ago |
| Water-filled pore space | WFPS_0.05.r-mean6+18 | WFPS in 5 cm depth (%), 3-hour mean ending 9 hours ago |
| Water-filled pore space | WFPS_0.15.r-mean6+6 | WFPS in 15 cm depth (%), 3-hour mean ending 3 hours ago |
| Water-filled pore space | WFPS_0.15.r-mean6+12 | WFPS in 15 cm depth (%), 3-hour mean ending 6 hours ago |
| Water-filled pore space | WFPS_0.15.r-mean6+18 | WFPS in 15 cm depth (%), 3-hour mean ending 9 hours ago |
| Water-filled pore space | WFPS_0.30.r-mean6+6 | WFPS in 30 cm depth (%), 3-hour mean ending 3 hours ago |
| Water-filled pore space | WFPS_0.30.r-mean6+12 | WFPS in 30 cm depth (%), 3-hour mean ending 6 hours ago |
| Water-filled pore space | WFPS_0.30.r-mean6+18 | WFPS in 30 cm depth (%), 3-hour mean ending 9 hours ago |
| Soil temperature | TS_0.05.r-mean6+6 | TS in 5 cm depth (°C), 3-hour mean ending 3 hours ago |
| Soil temperature | TS_0.05.r-mean6+12 | TS in 5 cm depth (°C), 3-hour mean ending 6 hours ago |
| Soil temperature | TS_0.05.r-mean6+18 | TS in 5 cm depth (°C), 3-hour mean ending 9 hours ago |
| Soil temperature | TS_0.15.r-mean6+6 | TS in 15 cm depth (°C), 3-hour mean ending 3 hours ago |
| Soil temperature | TS_0.15.r-mean6+12 | TS in 15 cm depth (°C), 3-hour mean ending 6 hours ago |
| Soil temperature | TS_0.15.r-mean6+18 | TS in 15 cm depth (°C), 3-hour mean ending 9 hours ago |
| Soil temperature | TS_0.30.r-mean6+6 | TS in 30 cm depth (°C), 3-hour mean ending 3 hours ago |
| Soil temperature | TS_0.30.r-mean6+12 | TS in 30 cm depth (°C), 3-hour mean ending 6 hours ago |
| Soil temperature | TS_0.30.r-mean6+18 | TS in 30 cm depth (°C), 3-hour mean ending 9 hours ago |
| Precipitation | PREC_r-mean6 | precipitation (mm), 3-hour mean over preceding 3 hours |
| Precipitation | PREC_r-mean6+6 | precipitation (mm), 3-hour mean ending 3 hours ago |
| Precipitation | PREC_r-mean6+12 | precipitation (mm), 3-hour mean ending 6 hours ago |
| Precipitation | PREC_r-mean6+18 | precipitation (mm), 3-hour mean ending 9 hours ago |
